# Self-guided digital treatment with virtual reality for panic disorder and agoraphobia: a study protocol for a randomized controlled trial

**DOI:** 10.1186/s13063-022-06366-x

**Published:** 2022-05-21

**Authors:** Jari Planert, Alla Machulska, Anne-Sophie Hildebrand, Kati Roesmann, Esra Otto, Tim Klucken

**Affiliations:** Department of Clinical Psychology and Psychotherapy, University of Siegen, Obergraben 23, 57072 Siegen, Germany

**Keywords:** Virtual reality, Cognitive behavioral therapy, Agoraphobia, Panic disorder, Self-guided help, Short-term treatment, E-health

## Abstract

**Background:**

Cognitive behavioral therapy is the first-line treatment for patients with panic disorder (PD) and agoraphobia (AG). Yet, many patients remain untreated due to limited treatment resources. Digital self-guided short-term treatment applications may help to overcome this issue. While some therapeutic applications are already supported by health insurance companies, data on their efficacy is limited. The current study investigates the effect of self-guided digital treatment comprising psychoeducation and virtual reality exposure therapy (VRET).

**Methods:**

Thirty patients diagnosed with PD, AG, or panic disorder with agoraphobia (PDA) will be randomly assigned to either the experimental group (EG) or the control group (CG). Participants of both groups will undergo baseline diagnostics in the first two sessions. The subsequent treatment for the EG consists of a self-guided 6-week phase of application-based psychoeducation, one therapy session preparing for the VRET, and 4 weeks of application-based self-guided VRET. To control for the potential effects of the therapy session with the therapist, the CG will receive relaxation and stress-reduction training instead. All patients will then undergo a closing session which terminates with the post-assessment (~ 10 weeks after baseline assessment) and a follow-up assessment 6 weeks following the closing session. Symptom severity (primary outcome) will be assessed at baseline, interim, post-treatment, and follow-up. Additionally, remission status (secondary outcome) will be obtained at follow-up. Both measures will be compared between the groups.

**Discussion:**

The current study aims at providing insights into the efficacy of short-term treatment applications including psychoeducation and self-guided VRET. If successful, this approach might be a feasible and promising way to ease the burden of PD, AG, and PDA on the public health system and contribute to a faster access to treatment.

**Trial registration:**

ISRCTN ISRCTN10661970. Prospectively registered on 17 January 2022.

**Supplementary Information:**

The online version contains supplementary material available at 10.1186/s13063-022-06366-x.

## Background

Anxiety disorders are among the most common mental health disorders worldwide and are associated with immense psychological burdens and psychosocial impairment. Among those, panic disorder (PD), agoraphobia (AG), and panic disorder with agoraphobia (PDA) make up for a great part of the diagnoses. The 12-month prevalence of PD, AG, and PDA across EU countries is approximately 2% [1]. However, research suggests that underdiagnosis in both disorders is common, indicating that the true number of affected individuals might be considerably higher [1, 2].

Both PD and AG are best characterized by immense fear that oftentimes leads to panic-like attacks [3]. According to the 10th edition of the International Statistical Classification of Diseases and Related Health Problems (ICD-10) [4, 5], patients with PD suffer from the reoccurrence of sudden and unexpected panic attacks, during which they experience a plethora of physiological symptoms (i.e., racing heartbeat, dyspnea) and an acute fear of death. Patients with AG do not suffer from unexpected panic attacks but they experience panic-like fear reactions in inescapable situations or when access to help is limited [6]. PD and AG frequently overlap, resulting in the classified diagnosis of PDA [7]. In this pattern, patients not only experience recurrent unexpected panic attacks, but also avoid places and situations in which panic attacks might occur [8]. Consequences are among other things social isolation, disability to work or pursue personal interests, and an increased risk for the onset of comorbidities, such as substance use disorder or depression [1, 7]. A core pathogenic mechanism of all three disorders is persistent avoidance behavior, which is maintained by the momentary relief experienced upon avoidance of and flight from feared situations [9]. Subsequently, activities are avoided that have previously been associated with panic attacks or panic-related body symptoms, such as doing sports or drinking coffee. As the course of the disorder progresses, avoidance behavior often generalizes from single stimuli to broad domains of daily life [10, 11].

Aiming at resolving the dysfunctional avoidance patterns, cognitive behavioral therapy (CBT) interventions are among the most effective treatment options for PD, AG, and PDA [12, 13]. Using a variety of interventions comprising psychoeducation, cognitive reappraisal, and exposure, patients are encouraged to reflect on and modify behavioral and cognitive patterns involved in the onset and maintenance of fear and panic attacks [8]. Especially exposure therapy has been found effective in the treatment of PD, AG, and PDA as it directly tackles the patients’ avoidance behavior [14]. For AG, exposure therapy consists of encountering anxiety-evoking situations, such as going on a bus or to the shopping center [14]. On the other side, during exposure therapy for PD, fear reactions are provoked by evoking somatic symptoms typically experienced throughout a panic attack, for instance by running on a treadmill or shaking the head in order to help the patient re-evaluate the experienced symptoms as non-threatening. This practice is also referred to as interoceptive exposure [15]. Despite its proven efficiency, a considerable part of patients cannot access exposure therapy, because treatment capacities are limited [16]. Many patients are left behind on long waiting lists, implying immense direct and indirect costs for both the person in need and society [17–19]. Given the heavy consequences of untreated PD, AG, and PDA, there is a pressing urge to increase the accessibility of psychotherapeutic support [7, 20, 21].

E-health interventions might be a promising tool to overcome this barrier of limited accessibility [22]. In particular, by developing smartphone applications that comprise guided self-aid, public health companies tried to address the outstanding shortage of therapy spots [23, 24]. Multiple studies support this approach, as it not only potentially makes mental health services accessible for an extensive bandwidth of people, but also applies proven elements of traditional CBT treatment by providing psychoeducation and self-insight on a low threshold level [25–27]. To increase the applicability of E-health for PD, AG, and PDA, it is suggested that developers and researchers need to focus on the implementation of new mechanisms and elements such as virtual reality (VR) in digital treatment interventions [28].

It has been shown that exposure is an effective treatment for PD, AG, and PDA [29–31]. However, due to time and monetary constraints, exposure is rarely offered during treatment [32]. VR technology is a promising tool to address this issue [33, 34]. By providing the possibility to encounter fear-provoking situations or sensations that are otherwise avoided while at the same time being in a safe environment, VR-based exposure therapy can be carefully executed step by step, responding to the client’s progress. As well as in vivo exposure therapy, virtual reality exposure therapy (VRET) differs between the disorders AG and PD [14]. For AG, virtual fear-evoking situations are simulated, in which the patient can learn to overcome their fear. For PD (without agoraphobia), on the other hand, interoceptive exposure is realized outside the VR [15]. Especially for anxiety disorders like PD and AG, VR is highly efficacious in both the short-term and long-term treatment settings [23, 35]. Also, in vivo exposure therapy has sometimes been the target of criticism due to lower acceptance rates as compared to other treatment approaches among patients [36]. VRET, in contrast, might be more patient-friendly than in vivo exposure, because the degree of exposure can be adjusted [37].

To this point, access to psychotherapy was often hindered by barriers in the mental health care system [17]. E-health interventions bridging this gap have been found effective, yet still need adjustments to find their way into clinical practice for the treatment of PD, AG, and PDA more frequently. One way to address the aforementioned challenge is a multilayered approach that comprises psychotherapeutic supervision in combination with a self-guided short-term treatment featuring VR elements. This digital short-term treatment option for PD, AG, and PDA is already supported by major health insurance companies that pay for the application as well as for the associated VR equipment [38]. This is a commendable attempt to help patients by overcoming bureaucratic obstacles. Yet, research is lacking on the efficacy of self-guided short-term treatments with VR elements. To close that gap, the present randomized controlled trial will be conducted to investigate the effects of a self-guided short-term treatment on the psychological symptoms in patients affected by PD, AG, and PDA.

### Trial design

The current study illustrates a single-centered randomized controlled trial to investigate whether self-guided short-term treatments via smartphone applications are a viable treatment method for PD, AG, and PDA. Eligible participants will be randomly assigned to either the experimental condition (2 diagnostic sessions + application-based psychoeducation + therapy session + self-guided VR-treatment + closing session) or the control (2 diagnostic sessions + therapy session + closing session). A 2 (condition: treatment vs. active control) × 4 (time: baseline/interim/post-treatment/follow-up) mixed design will be applied. As this study aims at contributing to better care for patients without access to regular treatment, we decided to include an active control group as a comparator, which controls for up to five sporadic consultation-hour sessions with a therapist. First, we expect that patients in the experimental condition will experience a significantly higher reduction of symptom severity than patients in the control condition during the interim, post-treatment, and follow-up measures, compared to the baseline assessment. The secondary hypothesis is that at follow-up, significantly fewer patients in the experimental condition will meet the diagnostic criteria for PD, AG, and PDA, compared to the control condition.

To estimate the required sample size, an a priori power analysis was conducted using G*Power 3.1.9.7 [39]. The primary hypothesis is tested using 2 × 4 mixed design repeated measures analyses of variance (ANOVA). The required total sample size to detect a moderate effect for a repeated measures ANOVA with within-between interaction (effect size *f* = .25) is *N* = 24, given an *α* level of .05 and achieved power of 1−*β* = .80. The effect size was inferred from previously reported effect sizes in the literature [23, 40, 41]. The assumed correlation between the repeated measures was set to .5. As the treatment includes digital treatment components, a drop-out rate of approximately 20% is expected, based on prior research [42]. Due to this expected attrition between the measurements, it is planned to include 30 patients, resulting in *N* = 15 per condition. The secondary hypothesis will be tested using the chi-square statistic.

## Methods

### Participants

Subjects will be approached via flyers, radio, television, newspaper, and online advertisements. The inclusion criteria will comprise the ICD-10 diagnostic criteria for agoraphobia, unspecified (F40.00), agoraphobia with panic disorder (F40.01), agoraphobia without panic disorder (F40.02), or panic disorder (F41.0) [5]. Additionally, participants need to be at least 18 years old to be eligible for taking part in this study. Based on contraindications as defined for the treatment application, the exclusion criteria will include the following conditions: stroke or myocardial infarction in patient history, angina pectoris, cardiac dysrhythmia, asthma, chronic obstructive pulmonary disease, pregnancy or assumed pregnancy, severely impaired vision, epilepsy, or other cramp attacks in patient history, a psychological disorder with an organic origin, dizziness or vestibular impairment, a psychological disorder due to the use of psychoactive substances, schizophrenia, schizotypal or delusional disorder, severe depression, acute suicidality, or missing agreement in the presence of suicidality [38].

Also, patients cannot be included if they already are in psychotherapeutic or psychopharmacological treatment containing the use of tricyclic antidepressants, monoamine oxidase inhibitors, selective serotonin reuptake inhibitors, and serotonin and norepinephrine reuptake inhibitors. Patients that previously used benzodiazepines as an acute medication are instructed to not use them for the time of the trial, if possible.

### Ethics statement

The present study was approved by the Ethical Committee of the University of Siegen (date of approval: 04-11-2021, reference number ER_48_2021). The study protocol is in line with the Declaration of Helsinki, the guidelines for Good Clinical Practice, and the SPIRIT reporting guidelines [43]. Before study inclusion, participants must provide written informed consent, which they can withdraw at any time without any adverse consequences. All obtained data will be processed pseudonymously in order to protect the confidentiality agreement before, during, and after the trial.

Participation in the study brings some minor risks for the patients, about which they will be informed before entering the trial. As the current trial partly consists of a self-guided intervention, some dangers of self-guided treatment need to be taken under careful consideration. One possible risk is that patients who do not benefit from the treatment may be less receptive to further psychotherapeutic interventions [44]. Another potential risk associated with the use of VR equipment is cybersickness. The VR modules of the treatment application consist of high-quality, short-term, pre-recorded 360° videos, which do not include fast motions or changes in frame rate and in which the participant is not required to move around. Following previous research on conditions evoking cybersickness, it should not be a concern for the current trial [45, 46]. For patients in the control condition, a potential risk is the occurrence of relaxation-induced anxiety or panic attacks during or after the third treatment session [47]. To measure potential side effects associated with the conduction of the study, patients will be asked about the occurrence of adverse events (e.g., cybersickness, relaxation-induced panic attacks) and whether these were associated with specific treatment elements during the fourth psychotherapeutic session of the corresponding research condition.

During the digital treatment, suicidality checks are conducted through the treatment application. In case of acute suicidality at any point of the treatment, the treating therapist will be notified immediately, and then decides if the treatment will be discontinued if required. If needed, more intensive care will be arranged such as the admission to an inpatient clinic.

### Procedure

**Fig. 1:**
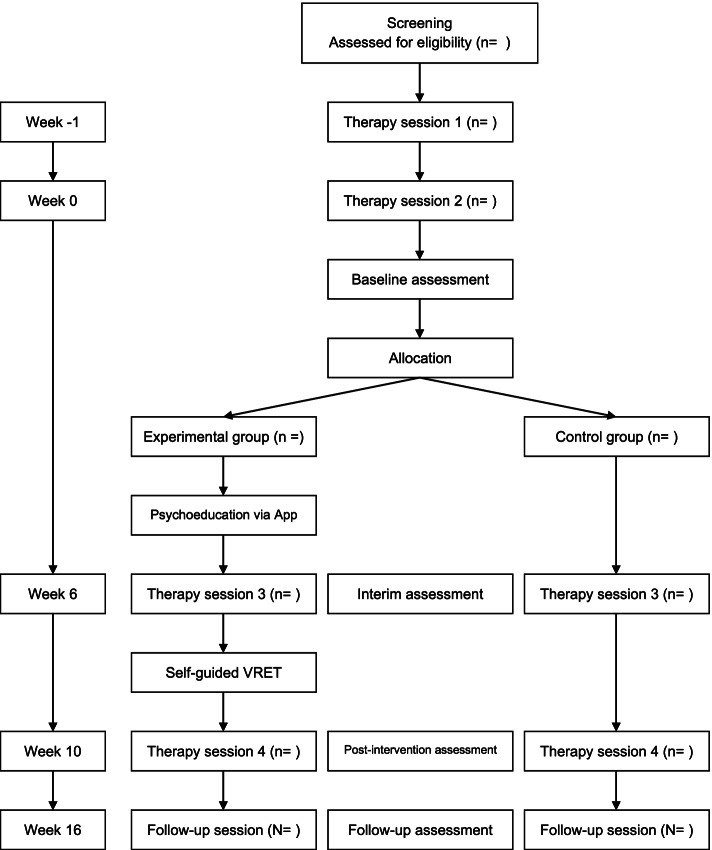
The planned patient contact protocol as a CONSORT flow diagram. The CONSORT flow diagram displays the undertaken steps from recruitment to follow-up diagnostics. At four points in time, outcome measures will be assessed. Week 0 indicates the time point of baseline diagnostics

The trial will take approximately 17 weeks for each patient to complete (see Fig. 1 for the planned patient contact protocol). After being informed via mail about the study’s content, interested participants will engage in a telephone interview to determine whether they fulfill the inclusion criteria. Following the screening, which will take 10–30 min, patients will be invited to the psychotherapeutic outpatient center of the University of Siegen for the first session, during which their symptoms will be assessed by a licensed psychotherapist. This first intake session is scheduled to take 50 min. If patients meet the requirements, they will be invited for a second 100-min intake session for baseline diagnostics, 1 week after the first intake. After the second intake session, patients will be allocated to the research conditions. At this point, participants in the experimental condition will receive access to the psychoeducational part of the treatment application. Six weeks later, the third session (150 min) will take place as a preparation session for the experimental group (EG), while the control group (CG) will receive relaxation training in the meantime. Then, patients in the EG will work on their own with the VRET of the treatment application for 4 weeks. After the intervention, patients will be invited for a closing session, which is scheduled to take 100 min. For both groups, the purpose of this session will be to reflect on the treatment process and to consolidate learned behavior in the context of the daily lives of the patients and for relapse prevention. Follow-up diagnostics will be performed 6 weeks after the closing session. At this point, patients in the control condition will receive access to the digital training as well. Patients that still meet the diagnostic criteria after the trial will be informed and advised on further treatment possibilities. If important modifications to the study protocol are needed, relevant parties will be informed by the corresponding author.

### Randomization and blinding

For the first two appointments of the study schedule, patients, therapists as well as research assistants involved in the data collection, preparation, and scoring will be blind to the research conditions. After the second appointment, patients will be randomly assigned to either the EG or the CG with a ratio of 1:1. The therapist will then receive a sealed envelope disclosing the patient’s allocation. The allocation scheme was generated before study initiation using R.4.1.4. Research assistants will remain blind to the patients’ group allocations. Follow-up diagnostics will be conducted by a licensed therapist who has not seen the patient beforehand and who will thus be blind to the treatment condition of this certain patient. However, complete blinding cannot be realized for therapists and patients due to the study design. Patients will be informed about the existence of two research conditions before the start of the trial as part of the telephone screening. Depending on whether they receive the prescription for the digital treatment application after the second session or not, they will be aware of their treatment condition. To minimize the effects of biases on the results, the statistician conducting the data analysis will be blinded to the research conditions.

### Intervention

After allocation to the research conditions, patients in the EG will receive access to the psychoeducational part of the self-guided treatment application, which they will be instructed to complete before the third session. The third session will be a therapy session (150 min), during which the patients allocated to the (EG) will be prepared for the self-guided VRET. Here, the patients are introduced to the rationale behind exposure therapy, they are guided through an anxiety-evoking situation in imagination by their therapist, and lastly, they learn to reevaluate their anxiety-evoking symptoms. In order to ensure that the psychotherapeutic contact is balanced out between the research conditions, patients in the CG will receive relaxation training instead, which is also scheduled as a manualized therapy session (150 min). The relaxation training consists of psychoeducation of the human stress physiology regarding their anxiety and of progressive muscle relaxation (PMR). The PMR will take approximately 40 min and is guided by the treating therapist. Then, while patients in the experimental condition will have 4 weeks to operate with the self-guided smartphone application, patients in the CG will be instructed to continue with PMR on their own. After that period, all patients will be invited for the fourth appointment, which consists of a closing session with a psychotherapist and a post-treatment assessment. One follow-up session will take place 6 weeks after the closing session.

### Self-guided digital treatment application with virtual reality elements

The self-guided digital short-term treatment application was developed to support the treatment of various anxiety disorders such as PD, AG, and PDA [38]. It consists of eight modules in total, of which the first six will be available upon the start of the treatment, including psychoeducation and cognitive techniques. For example, patients will be first introduced to key factors involved in the onset and maintenance of their anxiety and fear-related symptoms. As the treatment progresses, patients will be asked to apply their new knowledge by reflecting on their very own behavioral patterns (e.g., behavioral avoidance, safety signals) and experiences.

The remaining modules will be unlocked by the therapist after the third appointment in the outpatient clinic. In module 6, patients will face their anxiety: Self-guided VRET will be realized using a head-mounted construction allowing them to use their smartphone as a display. Additionally, headphones will be provided. For safety reasons, patients are asked to remain seated for the whole duration of the particular exposure session, while high-definition pre-recorded 360° videos are shown. Exposure scenarios include a subway ride during rush hour, being in an elevator or a crowd, driving on the highway, or driving in an open parking lot. Patients can look around freely; however, they cannot interact with or move within the displayed environment. In the treatment of PD, self-guided interoceptive exposure is added, to facilitate a reevaluation of somatic sensations as non-threatening. Some of these exercises require the patient to stand up and, for example, run on the spot. Therefore, these exercises are conducted without the head-mounted display, only using headphones. In the last two modules, patients will be asked to reflect on the treatment progress and on potential achievements they made.

### Material and measures

**Fig. 2:**
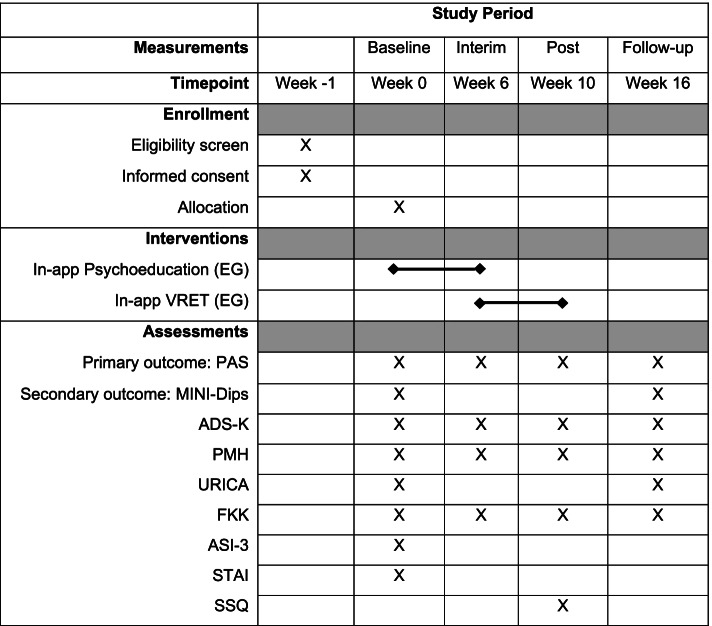
Planned measurement schedule. Schedule of all outcome measures regarding the different time points of the study protocol. An X in the corresponding box indicates that a measurement or an action takes place at a certain time point. Week − 1 indicates the first appointment. Week 0 marks the allocation of the patients to the research groups, from which point the EG receives psychoeducation. The follow-up takes place 6 weeks after the intervention has ended

The primary outcome measure will be the PD and AG symptom severity, as measured by the German version of the Panic and Agoraphobia Scale (PAS) [48]. The measure will be carried out at baseline, interim, post-interventional, and 6 weeks following the treatment (see Fig. 2 for the planned measurement schedule). The PAS consists of 13 items that measure panic attacks, agoraphobic avoidance, anticipatory anxiety, disability, and functional avoidance. Each item can be answered on a 5-point Likert scale. The internal consistency of the German version is good with a Cronbach’s alpha of .86 [48].

For the secondary outcome measure remission, the MINI-Dips will be used to assess whether patients meet the diagnostic criteria for PD, AG, or PDA. The MINI-Dips will be carried out at baseline and the follow-up [49]. For the secondary outcome measure clinically significant change of symptoms (CSC), the predefined cutoff score for the remission of the PAS will be used. As the total score can range from 0 to 40 and remission is considered at values between 0 and 8, an individual decline of symptoms of 80% is regarded as a CSC.

As the present study is conducted in cooperation with an outpatient clinic, additional outcome variables will routinely be measured and included in exploratory analyses. These measures will include general depression symptoms, measured through the German version of the General Depression Scale (ADS-K) [50]; general psychological well-being, as measured by the Positive Mental Health Scale (PMH) [51]; willingness to change, as measured by the German version of the University of Rhode Island Change Assessment Scale (FEVER) [52]; competency and locus of control, as measured by the German Competency and Locus of Control Questionnaire (FKK) [53]; anxiety sensitivity, as measured by the German version of the Anxiety Sensitivity Index-3 (ASI-3) [54]; state-trait anxiety, as measured by the German version of the State-Trait Anxiety Questionnaire (STAI) [55]; and cybersickness, as measured by the German version of the Simulator Sickness Questionnaire (SSQ) [56]. Additionally, to monitor adherence to the protocol, it will be measured if and how often the treatment application is accessed.

A data monitoring committee (DMC) will keep track of the data collection. It consists of student assistants involved in the data collection and the primary investigators. The DMC is independent of any third party such as the sponsor; none of its members is associated with the developer of the treatment application in any form.

### Data preparation and planned analysis

Multiple imputation based on demographic data will be used to replace missing data, once patients have completed at least the third psychotherapeutic session [57]. Data from patients that discontinued the treatment before randomization or right after the second session will be excluded from the analysis. Based on the intention-to-treat (ITT) principle, non-compliance to the treatment or deviation from the treatment plan will not lead to the exclusion of participants [58]. Outliers will be included in the analysis unless they indicate impossible values. To test whether randomization succeeded, baseline group differences of the variables age and gender, as well as baseline scores of the PAS, will be analyzed using *t*-tests for independent samples. Mauchly’s sphericity test will be conducted to monitor whether violations of the sphericity assumption occurred. If violated, the Greenhouse-Geisser correction (*ε* < .75) or the Hyunh-Feldt correction (*ε* > .75) will be applied, depending on the degree of violation [59]. To test the primary hypothesis, a 2 (condition: VR-intervention/active control) × 4 (time: baseline/interim/post-treatment/follow-up) repeated measures ANOVA will be conducted with the primary outcome measurement assessing panic and agoraphobia symptomology, the PAS. Here, the interaction *p*-value and effect size *R*^2^ will be interpreted. In case of a significant interaction effect, simple contrasts will be estimated to assess the differences between EG and CG across the specific time points. For the dichotomous clinical outcome measure remission at follow-up, a chi-square test of independence will be conducted. Another chi-square test of independence will be performed for the dichotomous CSC at follow-up.

## Discussion

PD, AG, and PDA are a heavy burden for the individual and society. Psychotherapy is effective, yet the access is limited, resulting in long waiting lists and aggravation of impairment. Digital treatment options are promising, but more research is needed to facilitate the application in a clinical context. The present trial, therefore, is designed to investigate the efficacy of a self-guided digital short-term treatment with VR-based exposure for PD, AG, and PDA. Combining self-report measures for symptom severity with a structured clinical interview performed by a licensed therapist, the results of this study can shed light on the issue of whether a cost-efficient self-aid treatment with minimal therapeutic supervision can lead to a decrease in PD, AG, and PDA symptoms and recovery.

The results of the present randomized controlled trial will provide new information about the efficacy of self-guided VR applications. Shedding light on the efficacy of such interventions is important to contextualize self-guided VR treatment as a possible treatment method for PD, AG, and PDA. If proven effective, self-guided interventions could constitute a cost-effective and time-efficient adjunction to existing traditional treatments.

The findings of the present study will provide important cues for clinical practice and future research. If the self-guided VR treatment is found to be effective, this trial can pave the way for a new wave of self-guided treatments to be integrated into the course of patient-centered therapy. This would lower the burden on the public mental health system and, more importantly, people would have to spend less time on long waiting lists before they can get access to the treatment they need. Once this new form of treatment is found to be effective, future research can take a step further to directly compare the self-guided VR treatment to a classic in vivo CBT treatment or whether the efficacy of CBT treatments could be enhanced by adding self-guided interventions to the treatment. To maximize the clinical applicability, future research could tackle the generalizability of potential findings by discovering if there are patient groups that benefit most from this digital treatment method.

### Trial status

The current trial was prospectively registered in the ISRCTN registry for current-controlled trials on 17 January 2022 (trial ID: ISRCTN10661970, protocol version number 1.0). Data collection has started in March 2022 and will be ended by March 2024 at the latest.

## Supplementary Information

**Additional file 1.**

## Data Availability

The dataset generated in the current study will be available from the corresponding author on reasonable request.

## Abbreviations

CBT: Cognitive behavioral therapy
PD: Panic disorder
AG: Agoraphobia
PDA: Panic disorder with agoraphobia
VR: Virtual reality
VRET: Virtual reality exposure therapy
EG: Experimental group
CG: Control group
ICD-10: International Statistical Classification of Diseases and Related Health Problems
PMR: Progressive muscle relaxation
PAS: Panic and Agoraphobia Scale
ADS-K: General Depression Scale
PMH: Positive Mental Health Scale
FEVER: University of Rhode Island Change Assessment Scale
FKK: Competency and Locus of Control Questionnaire
ASI-3: Anxiety Sensitivity Index-3
STAI: State-Trait Anxiety Questionnaire
SSQ: Simulator Sickness Questionnaire
ANOVA: Analysis of variance
